# Supplementary material for: Precautionary Health Behaviours as Potential Confounders in COVID-19 Vaccine Effectiveness Studies
Source: Vaccines (Basel). 2025 Oct 12;13(10):1047. doi: 10.3390/vaccines13101047 (PMC12567800; doi:10.3390/vaccines13101047)
Supplement: Supplementary file 1 [file vaccines-13-01047-s001.zip › Supplementary figure PHB.pdf]

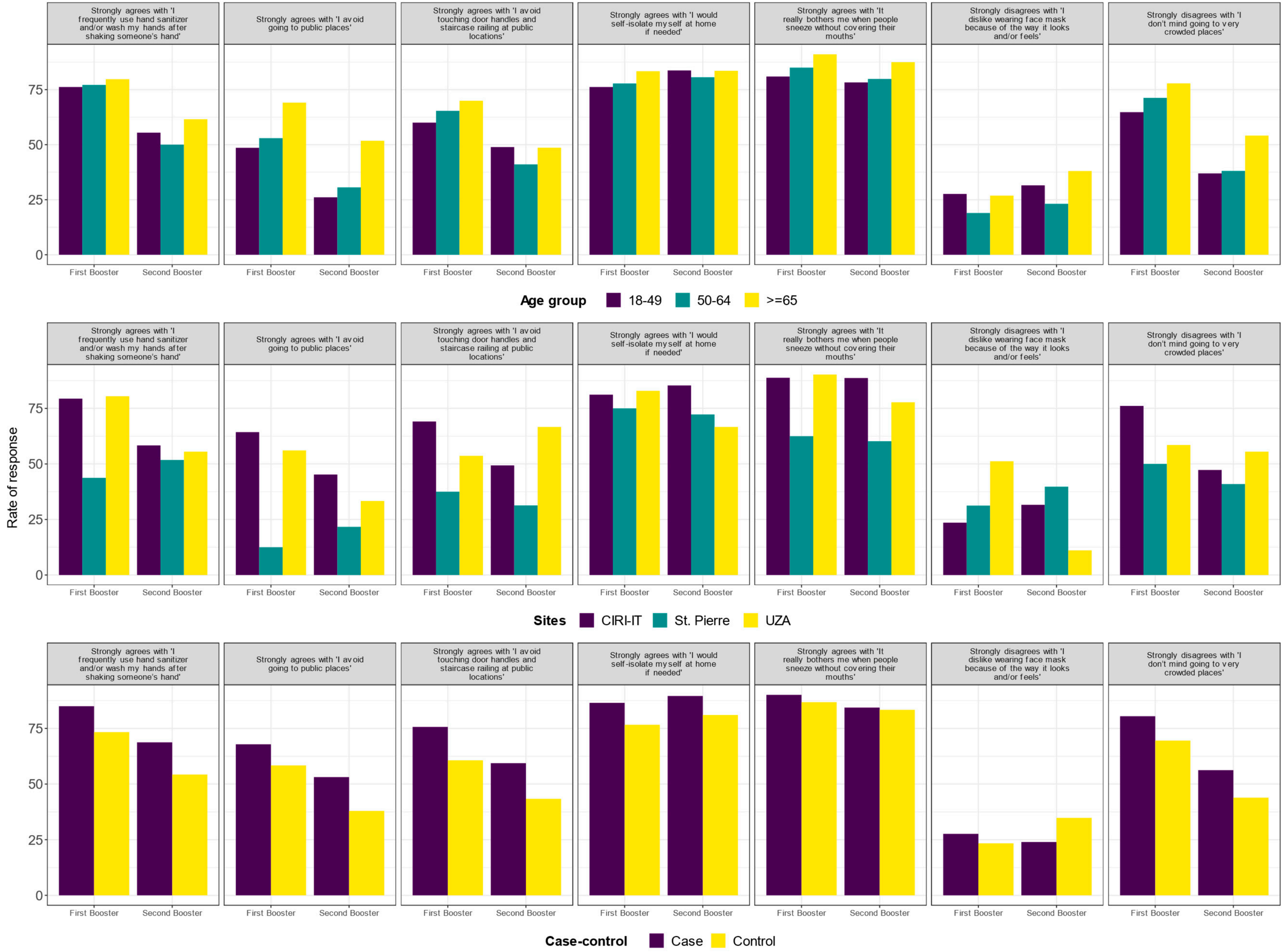
Strongly agrees with 'I frequently use hand sanitizer and/or wash my hands after shaking someone's hand'
Strongly agrees with 'I avoid going to public places'
Strongly agrees with 'I avoid touching door handles and staircase railing at public locations'
Strongly agrees with 'I would self-isolate myself at home if needed'
Strongly agrees with 'It really bothers me when people sneeze without covering their mouths'
Strongly disagrees with 'I dislike wearing face mask because of the way it looks and/or feels'
Strongly disagrees with 'I don't mind going to very crowded places'
0
25
50
75
First Booster
Second Booster
Rate of response
Age group
18-49
50-64
>=65
Sites
CIRI-IT
St. Pierre
UZA
Case-control
Case
Control

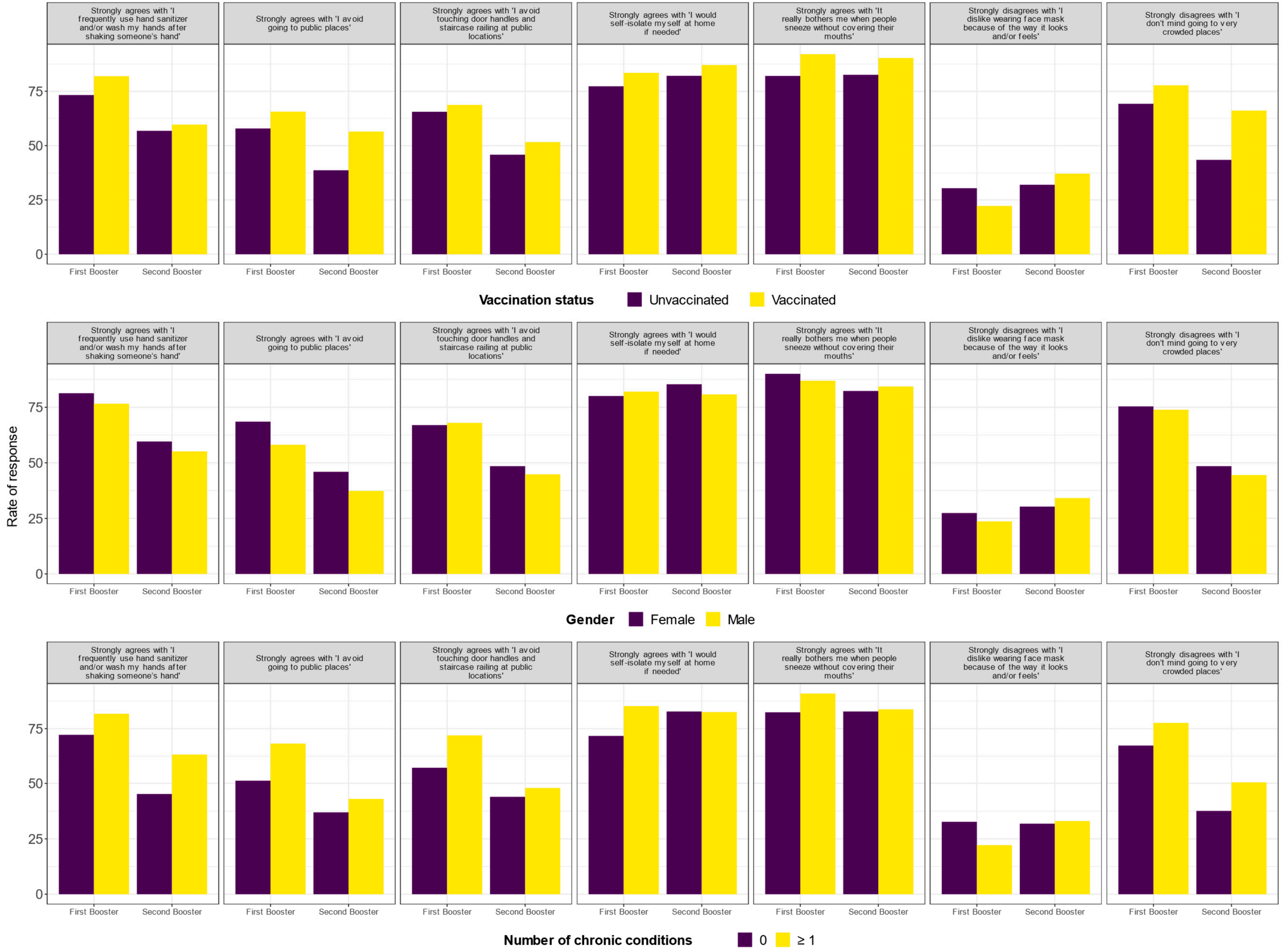
Strongly agrees with 'I frequently use hand sanitizer and/or wash my hands after shaking someone's hand'
Strongly agrees with 'I avoid going to public places'
Strongly agrees with 'I avoid touching door handles and staircase railing at public locations'
Strongly agrees with 'I would self-isolate myself at home if needed'
Strongly agrees with 'It really bothers me when people sneeze without covering their mouths'
Strongly disagrees with 'I dislike wearing face mask because of the way it looks and/or feels'
Strongly disagrees with 'I don't mind going to very crowded places'
First Booster
Second Booster
Vaccination status
Unvaccinated
Vaccinated
Gender
Female
Male
Number of chronic conditions
0
≥ 1
Rate of response
0
25
50
75

Figure S1: Percentages of participants with PHB indicators by age group and COVID-19 booster season.

Rate of response: percentage of participants with PHB indicator (statement meeting its highest score: strongly agree for statements indicative of PHB; strongly disagree for statements indicative of non-PHB).
