# Supplementary material for: Precautionary Health Behaviours as Potential Confounders in COVID-19 Vaccine Effectiveness Studies
Source: Vaccines (Basel). 2025 Oct 12;13(10):1047. doi: 10.3390/vaccines13101047 (PMC12567800; doi:10.3390/vaccines13101047)
Supplement: Supplementary file 1 [file vaccines-13-01047-s001.zip › Supplementary tables PHB.pdf]

Table S1: Outcomes of the survey indicative of precautionary health behaviours.

Survey outcome	Age N (%)			Sex N (%)		Chronic conditions N (%)		Sites N (%)			Case–control N (%)		Exposure status N (%)	
**First booster season**	**18–49 (N=105)**	**50–64 (N=153)**	**≥65 (N=469)**	**Female (N=321)**	**Male (N=406)**	**≥1 (N=510)**	**0 (N=205)**	**CIRI-IT (N=670)**	**UZA (N=41)**	**CHU St. Pierre (N=16)**	**Case (N=333)**	**Control (N=394)**	**Unvaccinated (N=273)**	**Vaccinated (N=454)**
**It really bothers me when people sneeze without covering their mouths**														
Strongly disagree	17 (16.2)	18 (11.8)	37 (7.9)	29 (9.0)	43 (10.6)	39 (7.6)	33 (16.1)	67 (10.0)	3 (7.3)	2 (12.5)	28 (8.4)	44 (11.2)	44 (16.1)	28 (6.2)
Undecided	3 (2.9)	5 (3.3)	5 (1.1)	3 (0.9)	10 (2.5)	7 (1.4)	3 (1.5)	8 (1.2)	1 (2.4)	4 (25.0)	5 (1.5)	8 (2.0)	5 (1.8)	8 (1.8)
Strongly agree	85 (81.0)	130 (85.0)	427 (91.0)	289 (90.0)	353 (86.9)	464 (91.0)	169 (82.4)	595 (88.8)	37 (90.2)	10 (62.5)	300 (90.1)	342 (86.8)	224 (82.1)	418 (92.1)
**I avoid touching door handles and staircase railing at public locations**														
Strongly disagree	36 (34.3)	44 (28.8)	119 (25.4)	90 (28.0)	109 (26.8)	119 (23.3)	75 (36.6)	180 (26.9)	11 (26.8)	8 (50.0)	66 (19.8)	133 (33.8)	82 (30.0)	117 (25.8)
Undecided	6 (5.7)	9 (5.9)	22 (4.7)	16 (5.0)	21 (5.2)	24 (4.7)	13 (6.3)	27 (4.0)	8 (19.5)	2 (12.5)	15 (4.5)	22 (5.6)	12 (4.4)	25 (5.5)
Strongly agree	63 (60.0)	100 (65.4)	328 (69.9)	215 (67.0)	276 (68.0)	367 (72.0)	117 (57.1)	463 (69.1)	22 (53.7)	6 (37.5)	252 (75.7)	239 (60.7)	179 (65.6)	312 (68.7)
**I would self-isolate myself at home if needed**														
Strongly disagree	19 (18.1)	23 (15.0)	56 (11.9)	42 (13.1)	56 (13.8)	51 (10.0)	43 (21.0)	90 (13.4)	5 (12.2)	3 (18.8)	32 (9.6)	66 (16.8)	47 (17.2)	51 (11.2)
Undecided	6 (5.7)	11 (7.2)	22 (4.7)	22 (6.9)	17 (4.2)	24 (4.7)	15 (7.3)	36 (5.4)	2 (4.9)	1 (6.2)	13 (3.9)	26 (6.6)	15 (5.5)	24 (5.3)
Strongly agree	80 (76.2)	119 (77.8)	391 (83.4)	257 (80.1)	333 (82.0)	435 (85.3)	147 (71.7)	544 (81.2)	34 (82.9)	12 (75.0)	288 (86.5)	302 (76.6)	211 (77.3)	379 (83.5)
**I frequently use hand sanitizer and/or wash my hands after shaking someone's hand**														

Strongly disagree	17 (16.2)	24 (15.7)	75 (16.0)	45 (14.0)	71 (17.5)	68 (13.3)	45 (22.0)	108 (16.1)	3 (7.3)	5 (31.2)	38 (11.4)	78 (19.8)	58 (21.2)	58 (12.8)
Undecided	8 (7.6)	11 (7.2)	20 (4.3)	15 (4.7)	24 (5.9)	25 (4.9)	12 (5.9)	30 (4.5)	5 (12.2)	4 (25.0)	12 (3.6)	27 (6.9)	15 (5.5)	24 (5.3)
Strongly agree	80 (76.2)	118 (77.1)	374 (79.7)	261 (81.3)	311 (76.6)	417 (81.8)	148 (72.2)	532 (79.4)	33 (80.5)	7 (43.8)	283 (85.0)	289 (73.4)	200 (73.3)	372 (81.9)
**I avoid going to public places**														
Strongly disagree	49 (46.7)	62 (40.5)	118 (25.2)	81 (25.2)	148 (36.5)	133 (26.1)	88 (42.9)	208 (31.0)	11 (26.8)	10 (62.5)	94 (28.2)	135 (34.3)	98 (35.9)	131 (28.9)
Undecided	5 (4.8)	10 (6.5)	27 (5.8)	20 (6.2)	22 (5.4)	29 (5.7)	12 (5.9)	31 (4.6)	7 (17.1)	4 (25.0)	13 (3.9)	29 (7.4)	17 (6.2)	25 (5.5)
Strongly agree	51 (48.6)	81 (52.9)	324 (69.1)	220 (68.5)	236 (58.1)	348 (68.2)	105 (51.2)	431 (64.3)	23 (56.1)	2 (12.5)	226 (67.9)	230 (58.4)	158 (57.9)	298 (65.6)
**I dislike wearing face mask because of the way it looks and/or feels**														
Strongly disagree	29 (27.6)	29 (19.0)	126 (26.9)	88 (27.4)	96 (23.6)	113 (22.2)	67 (32.7)	158 (23.6)	21 (51.2)	5 (31.2)	92 (27.6)	92 (23.4)	83 (30.4)	101 (22.2)
Undecided	5 (4.8)	10 (6.5)	31 (6.6)	26 (8.1)	20 (4.9)	29 (5.7)	15 (7.3)	40 (6.0)	4 (9.8)	2 (12.5)	12 (3.6)	34 (8.6)	19 (7.0)	27 (5.9)
Strongly agree	71 (67.6)	114 (74.5)	312 (66.5)	207 (64.5)	290 (71.4)	368 (72.2)	123 (60.0)	472 (70.4)	16 (39.0)	9 (56.2)	229 (68.8)	268 (68.0)	171 (62.6)	326 (71.8)
**I do not mind going to very crowded places**														
Strongly disagree	68 (64.8)	109 (71.2)	365 (77.8)	242 (75.4)	300 (73.9)	396 (77.6)	138 (67.3)	510 (76.1)	24 (58.5)	8 (50.0)	268 (80.5)	274 (69.5)	189 (69.2)	353 (77.8)
Undecided	5 (4.8)	13 (8.5)	38 (8.1)	30 (9.3)	26 (6.4)	40 (7.8)	16 (7.8)	46 (6.9)	7 (17.1)	3 (18.8)	17 (5.1)	39 (9.9)	17 (6.2)	39 (8.6)
Strongly agree	32 (30.5)	31 (20.3)	66 (14.1)	49 (15.3)	80 (19.7)	74 (14.5)	51 (24.9)	114 (17.0)	10 (24.4)	5 (31.2)	48 (14.4)	81 (20.6)	67 (24.5)	62 (13.7)
**Survey outcome**	**Age N (%)**			**Sex N (%)**		**Chronic conditions N (%)**		**Sites N (%)**			**Case–control N (%)**		**Exposure status N (%)**	

Second booster season	18–49 (N=92)	50–64 (N=134)	≥65 (N=255)	Female (N=198)	Male (N=281)	≥1 (N=321)	0 (N=157)	CIRI-IT (N=389)	UZA (N=9)	CHU St. Pierre (N=83)	Case (N=96)	Control (N=385)	Unvaccinated (N=419)	Vaccinated (N=62)
**It really bothers me when people sneeze without covering their mouths**														
Strongly disagree	11 (12.0)	18 (13.4)	23 (9.0)	23 (11.6)	29 (10.3)	34 (10.6)	18 (11.5)	33 (8.5)	2 (22.2)	17 (20.5)	8 (8.3)	44 (11.4)	48 (11.5)	4 (6.5)
Undecided	9 (9.8)	9 (6.7)	9 (3.5)	12 (6.1)	15 (5.3)	18 (5.6)	9 (5.7)	11 (2.8)	0 (0.0)	16 (19.3)	7 (7.3)	20 (5.2)	25 (6.0)	2 (3.2)
Strongly agree	72 (78.3)	107 (79.9)	223 (87.5)	163 (82.3)	237 (84.3)	269 (83.8)	130 (82.8)	345 (88.7)	7 (77.8)	50 (60.2)	81 (84.4)	321 (83.4)	346 (82.6)	56 (90.3)
**I avoid touching door handles and staircase railing at public locations**														
Strongly disagree	36 (39.1)	56 (41.8)	89 (34.9)	74 (37.4)	107 (38.1)	121 (37.7)	58 (36.9)	135 (34.7)	1 (11.1)	45 (54.2)	25 (26.0)	156 (40.5)	159 (37.9)	22 (35.5)
Undecided	11 (12.0)	23 (17.2)	42 (16.5)	28 (14.1)	48 (17.1)	46 (14.3)	30 (19.1)	62 (15.9)	2 (22.2)	12 (14.5)	14 (14.6)	62 (16.1)	68 (16.2)	8 (12.9)
Strongly agree	45 (48.9)	55 (41.0)	124 (48.6)	96 (48.5)	126 (44.8)	154 (48.0)	69 (43.9)	192 (49.4)	6 (66.7)	26 (31.3)	57 (59.4)	167 (43.4)	192 (45.8)	32 (51.6)
**I would self-isolate myself at home if needed**														
Strongly disagree	11 (12.0)	17 (12.7)	26 (10.2)	23 (11.6)	31 (11.0)	34 (10.6)	20 (12.7)	40 (10.3)	1 (11.1)	13 (15.7)	7 (7.3)	47 (12.2)	51 (12.2)	3 (4.8)
Undecided	4 (4.3)	9 (6.7)	16 (6.3)	6 (3.0)	23 (8.2)	22 (6.9)	7 (4.5)	17 (4.4)	2 (22.2)	10 (12.0)	3 (3.1)	26 (6.8)	24 (5.7)	5 (8.1)
Strongly agree	77 (83.7)	108 (80.6)	213 (83.5)	169 (85.4)	227 (80.8)	265 (82.6)	130 (82.8)	332 (85.3)	6 (66.7)	60 (72.3)	86 (89.6)	312 (81.0)	344 (82.1)	54 (87.1)
**I frequently use hand sanitizer and/or wash my hands after shaking someone's hand**														
Strongly disagree	29 (31.5)	35 (26.1)	62 (24.3)	51 (25.8)	75 (26.7)	72 (22.4)	53 (33.8)	103 (26.5)	2 (22.2)	21 (25.3)	18 (18.8)	108 (28.1)	109 (26.0)	17 (27.4)
Undecided	12 (13.0)	32 (23.9)	36 (14.1)	29 (14.6)	51 (18.1)	46 (14.3)	33 (21.0)	59 (15.2)	2 (22.2)	19 (22.9)	12 (12.5)	68 (17.7)	72 (17.2)	8 (12.9)
Strongly agree	51 (55.4)	67 (50.0)	157 (61.6)	118 (59.6)	155 (55.2)	203 (63.2)	71 (45.2)	227 (58.4)	5 (55.6)	43 (51.8)	66 (68.8)	209 (54.3)	238 (56.8)	37 (59.7)

**I avoid going to public places**														
Strongly disagree	51 (55.4)	61 (45.5)	82 (32.2)	78 (39.4)	115 (40.9)	131 (40.8)	61 (38.9)	136 (35.0)	4 (44.4)	54 (65.1)	34 (35.4)	160 (41.6)	177 (42.2)	17 (27.4)
Undecided	17 (18.5)	32 (23.9)	41 (16.1)	29 (14.6)	61 (21.7)	52 (16.2)	38 (24.2)	77 (19.8)	2 (22.2)	11 (13.3)	11 (11.5)	79 (20.5)	80 (19.1)	10 (16.1)
Strongly agree	24 (26.1)	41 (30.6)	132 (51.8)	91 (46.0)	105 (37.4)	138 (43.0)	58 (36.9)	176 (45.2)	3 (33.3)	18 (21.7)	51 (53.1)	146 (37.9)	162 (38.7)	35 (56.5)
**I dislike wearing face mask because of the way it looks and/or feels**														
Strongly disagree	29 (31.5)	31 (23.1)	97 (38.0)	60 (30.3)	96 (34.2)	106 (33.0)	50 (31.8)	123 (31.6)	1 (11.1)	33 (39.8)	23 (24.0)	134 (34.8)	134 (32.0)	23 (37.1)
Undecided	25 (27.2)	36 (26.9)	50 (19.6)	40 (20.2)	71 (25.3)	66 (20.6)	45 (28.7)	83 (21.3)	4 (44.4)	24 (28.9)	26 (27.1)	85 (22.1)	98 (23.4)	13 (21.0)
Strongly agree	38 (41.3)	67 (50.0)	108 (42.4)	98 (49.5)	114 (40.6)	149 (46.4)	62 (39.5)	183 (47.0)	4 (44.4)	26 (31.3)	47 (49.0)	166 (43.1)	187 (44.6)	26 (41.9)
**I do not mind going to very crowded places**														
Strongly disagree	34 (37.0)	51 (38.1)	138 (54.1)	96 (48.5)	125 (44.5)	162 (50.5)	59 (37.6)	184 (47.3)	5 (55.6)	34 (41.0)	54 (56.2)	169 (43.9)	182 (43.4)	41 (66.1)
Undecided	22 (23.9)	39 (29.1)	55 (21.6)	47 (23.7)	69 (24.6)	66 (20.6)	50 (31.8)	97 (24.9)	3 (33.3)	16 (19.3)	19 (19.8)	97 (25.2)	103 (24.6)	13 (21.0)
Strongly agree	36 (39.1)	44 (32.8)	62 (24.3)	55 (27.8)	87 (31.0)	93 (29.0)	48 (30.6)	108 (27.8)	1 (11.1)	33 (39.8)	23 (24.0)	119 (30.9)	134 (32.0)	8 (12.9)

Abbreviations: CHU, Centre Hospitalier Universitaire; CIRI-IT, Centro Interuniversitario per la Ricerca sull'Influenza e le Altre Infezioni Trasmissibili; UZA, Antwerp University Hospital.

Table S2: Sensitivity analysis of COVID-19 VE estimates using GAM instead of GEE.

	COVID-19 VE, GAM reference model[a] % (95% CI)	COVID-19 VE, GAM PHB-adjusted model 1[b] % (95% CI)	Percentage change between GAM reference model[a] and PHB-adjusted model 1[b]	p-value*
**First booster season**	46.2 (21.6; 63.1)	54.4 (32.5; 69.2)	17.75%	0.037
**Second booster season**	33.3 (-45.4; 69.4)	37.3 (-37.2; 71.3)	12.01%	0.067

Abbreviations: CI, confidence interval; GAM, generalised additive models; PHB, precautionary health behaviours; VE, vaccine effectiveness; [a] GAM reference model: GAM-based model, adjusted for symptom-onset date, sex, age, and number of chronic conditions; [b]GAM PHB-Adjusted model 1: GAM reference model with the PHB composite score fitted as a continuous variable; *Likelihood-ratio test p-value. p-values < 0.05 indicate that, based on goodness-of-fit, the adjusted model should not be rejected

Table S3: Sensitivity analysis of COVID-19 VE estimates using different cut-offs for PHB-adjusted model 2.

	COVID-19 VE, GEE PHB-adjusted model 2[a], cut-off ≥4 % (95% CI)	Percentage change between GEE reference model[b] and PHB-adjusted model 1[b]	p-value*	COVID-19 VE, GEE PHB-adjusted model 2[a], cut-off ≥6 % (95% CI)	Percentage change between GAM reference model[b] and PHB-adjusted model 1[b]	p-value*
**First booster season**	51.1 (46.5; 55.4)	10.13%	<0,001	51.7 (46.5; 56.4)	11.42%	<0,001
**Second booster season**	34.7 (18.8; 47.5)	8.44 %	<0,001	37.4 (24.5; 48.1)	16.87%	<0,001

[a]GEE PHB-Adjusted model 2: GEE reference model with the PHB composite score fitted as a binary variable, using a cut-off of ≥4 or ≥6 as sensitivity analysis

[b]GEE PHB reference model: GEE-based model, adjusted for symptom-onset date, sex, age, and number of chronic conditions. Results are those of Table 3.

* Likelihood ratio test p-value. p-values < 0.05 indicate that, based on goodness-of-fit, the adjusted model should not be rejected

Abbreviations: CI, confidence interval; GEE, generalised estimating equation; PHB, precautionary health behaviours; VE, vaccine effectiveness.
