# Supplementary material for: Precautionary Health Behaviours as Potential Confounders in COVID-19 Vaccine Effectiveness Studies
Source: Vaccines (Basel). 2025 Oct 12;13(10):1047. doi: 10.3390/vaccines13101047 (PMC12567800; doi:10.3390/vaccines13101047)
Supplement: Supplementary file 1 [file vaccines-13-01047-s001.zip › Supplementary text PHB.pdf]

Text S1: Precautionary Health Behaviour self-assessment questionnaire.

During the last three months,
It really bothers me when people sneeze without covering their mouth
0= strongly disagree
1= undecided
2= strongly agree

I avoid touching door handles and staircase railings at public locations
0= strongly disagree
1= undecided
2= strongly agree

I would self-isolate myself at home if needed
0= strongly disagree
1= undecided
2= strongly agree

I frequently use hand sanitiser and/or wash my hands after shaking someone's hand
0= strongly disagree
1= undecided
2= strongly agree

I avoid going to public places
0= strongly disagree
1= undecided
2= strongly agree

I dislike wearing a face mask because of the way it looks and/or feels.
0= strongly agree
1= undecided
2= strongly disagree

I don't mind going to very crowded places
0= strongly agree
1= undecided
2= strongly disagree

Adapted from Iorfa et al. COVID-19 Knowledge, Risk Perception, and Precautionary Behaviour Among Nigerians: A Moderated Mediation Approach. Front Psychol. 2020 Nov 20;11:566773. doi: 10.3389/fpsyg.2020.566773.
